# Testing cardiovascular autonomic function in the COVID-19 era: lessons from Bologna’s Autonomic Unit

**DOI:** 10.1007/s10286-020-00710-4

**Published:** 2020-07-13

**Authors:** Pietro Guaraldi, Giorgio Barletta, Francesca Baschieri, Giovanna Calandra-Buonaura, Federica Provini, Pietro Cortelli

**Affiliations:** 1 IRCCS Istituto delle Scienze Neurologiche di Bologna, UOC Clinica Neurologica NeuroMet, Ospedale Bellaria, Via Altura 3, 40139 Bologna, Italy; 2 Dipartimento di Scienze Biomediche e Neuromotorie, Università di Bologna, Bologna, Italy

**Keywords:** COVID-19, SARS-CoV-2, Coronavirus, Autonomic nervous system, Autonomic units, Cardiovascular autonomic testing

## Abstract

The coronavirus disease 2019 (COVID-19) pandemic has changed the way most medical procedures are performed. Autonomic units, as well as other healthcare sectors, are required to undergo a thorough reorganization of the protocols in order to guarantee the safety of patients and healthcare staff. Cardiovascular autonomic function testing (CAFT) is necessary in certain situations; however, it poses several concerns which need to be addressed. Here, we provide some practical advice based on current national and international health authorities’ recommendations and our experience about how to perform CAFT during the COVID-19 emergency. We examine aspects regarding patients, healthcare staff, laboratory preparation, and test performance.

## Introduction

The coronavirus disease 2019 (COVID-19) pandemic has disclosed the unpreparedness of healthcare systems worldwide, which have been compelled to take drastic measures to reorganize their services over a very short period of time to face this medical emergency of unprecedented proportions. Even though the COVID-19 pandemic is hopefully expected to be temporary, it will change the way most medical procedures are performed.

At the beginning of the COVID-19 outbreak in Italy [1] one of the immediate actions taken in the healthcare sector was to postpone all non-urgent procedures to avoid overcrowding hospital facilities. Other reasons were the shortages of personal protective equipment (PPE) and the need to redistribute healthcare staff in COVID-19 dedicated units. In the meantime, strategies involving telehealth technologies were developed to allow the care of patients, especially those affected by chronic diseases, from their home environment.

As social distancing and lockdown measures are becoming effective and infection rates decrease, a gradual reinstating of routine medical activities is reasonable and advisable. The main concern is, obviously, to guarantee the safety of patients and healthcare workers.

Autonomic units are located only in main and central hospitals; however, these units provide several tests and serve patients referred even from remote and peripheral areas. Therefore, a thorough reorganization of the protocols is required in order to safely restart their activities [2]. One of the main services provided is cardiovascular autonomic function testing (CAFT), which is necessary in certain situations, especially for patients who manifest symptoms and signs of dysautonomia that cannot be clarified only with clinical examination [3].

The main obstacles are (a) performing CAFT requires close vicinity between the patients and healthcare staff; (b) size of the laboratory room; (c) parts of the equipment are not disposable; (d) some maneuvers imply forced breathing and therefore cannot be performed with the face mask.

Here, on the basis of the experience at our Institution, which is a tertiary referral center for autonomic disorders in northern Italy, and current national and international health authorities’ recommendations, we provide guidance to reorganize autonomic laboratories’ activities during the COVID-19 pandemic, in order to continue providing medical service while not putting at risk patients and healthcare workers. Considering the different impact of the COVID-19 pandemic between different nations and also regions within the same country, the proposed suggestions may be tailored according to local legislation. It should be kept in mind that current regulations and suggestions may vary according to the rapidly evolving scenario and new emerging scientific evidence.

## Practical advice

### Laboratory room preparation

The size of the laboratory must be at least 12–15 m^2^ in order to safely accommodate two people (patient and staff member).

All unnecessary equipment should be removed from the laboratory room. If the room is sufficiently large, storage cabinets might be kept as long as they are covered.

Ensure that all the remaining elements can be sanitized with the appropriate solutions according to institutional/national protocols [4].

Place colored tape to delineate the area within 1 m from the tilt table [5] and position all equipment (computers, monitors, furniture) outside this area.

Cover all the equipment that may touch the patient with a disposable sheet.

Clean and disinfect the room according to hospital protocols.

### Patient preparation

On the day before the examination, all patients should be contacted by telephone (telephone triage) [6]. Besides reviewing general advice regarding CAFT (indications regarding meals, medications, lifestyle), the patients must be interviewed about the occurrence of fever, cough, new and persistent shortness of breath that does not subside with lying down (so as to distinguish it from orthostatic dyspnea due to orthostatic intolerance), myalgias, acute olfactory and/or gustatory dysfunction, headache, sore throat, congestion or runny nose, nausea, vomiting and diarrhea [7]. If possible COVID-19 symptoms are present, the assessment should be postponed. Otherwise, the patient is instructed to come to the hospital wearing a medical face mask and to be accompanied by one person only. The telephone triage is particularly important considering that patients are also referred to our center from distant areas and travelling might contribute to the spread of COVID-19. In this regard, travel is subjected to national regulations and only patients that are allowed to travel are admitted to the hospital for testing. It is important to also screen companions of non-self-sufficient patients, as they might help during the test preparation and therefore might come close to the healthcare staff.

On the day of the assessment, upon arrival, the patient and the accompanying person are screened at the hospital main entrance to check for symptoms that could be related to COVID-19 and any potential risk factor associated with exposure or recent travel to affected areas [6]. Body temperature is also measured to screen for fever. The laboratory front desk also serves as a screening site, and a further and similar screening of the patient is performed. Patients presenting respiratory symptoms will not be allowed to undergo CAFT and will be asked to return to their homes and to refer to their primary care physician for appropriate treatment and follow-up.

In case of necessity to perform CAFT in a suspected or confirmed COVID-19 patient, the same protocol applies but with careful additional consideration regarding the risk–benefit of the procedures to be performed (see below).

### Healthcare staff preparation

Staff members are requested to check daily their health status and measure body temperature every time before getting to work. Staff members are also requested to stay at home if they experience fever and/or respiratory symptoms. This applies not only to personnel interacting with patients but also to fellow employees and those working on shared workstations.

Doctors, technicians, nurses, and all other members of the staff who work in or in the proximity of the laboratory must know and be compliant with the infection control rules in use at the hospital, as well as wear appropriate PPE.

Before starting CAFT, healthcare staff responsible for the execution of the tests will wear disposable surgical gown, cap, gloves, and face mask (FFP-2) with eye protection (shield or goggles) [8, 9]. Such equipment is required because during CAFT the patients perform maneuvers that have the potential to generate aerosol particles.

We have rescheduled the laboratory daily routine by reducing the number of exams to avoid patients overcrowding and also the workload of the personnel, allowing the possibility of taking breaks in a safe environment and removing part of the PPE. This is advisable considering that a high level of alertness will be required during all working hours to avoid possible errors in the infection control process. Furthermore, this is likely to reduce the discomfort associated with prolonged use of PPE [10].

By wearing appropriate PPE and adopting all the precautions (especially avoiding close contact) for all patients, we expect the risk of infection transmission to be minimized [11]. In case a patient recently tested in the laboratory is found to be infected (test performed within 48 h before the onset of symptoms or the collection of the sample that proved the COVID-19 infection), our hospital policy, in line with international guidelines [12], is to promptly test the healthcare personnel that had come in contact with the patient by means of nasal swab and coronavirus serum antibody titer.

### Selecting the battery of cardiovascular autonomic function tests

Our battery of CAFT includes head-up tilt table test, Valsalva maneuver, deep breathing test, cold-face test and isometric exercise test, which are performed according to standardized procedures [13]. This battery allows the assessment of both adrenergic and cardiovagal domains and provides a global and detailed evaluation of cardiovascular autonomic function.

At the present time, however, our priority is to select the tests with the best risk–benefit profile, i.e., which could be informative but with limited risk of spreading the infection. To begin with, we avoid monitoring oronasal breathing (which would require the use of a sensor placed under the nostrils or a nasal cannula) and do not routinely perform the cold-face test (which provides complementary information). Instead, it may be valuable to add to the standard equipment the recording from a pulse oximeter in order to detect possible desaturation in paucisymptomatic patients with COVID-19. Measurement of plasma catecholamines during tilt table test is necessary in certain situations to investigate the pathophysiology of neurogenic orthostatic hypotension. The placement of an indwelling catheter and drawing samples are additional procedures that require special caution. We record electroencephalogram (EEG) only when CAFT is performed for the differential diagnosis of episodes of transient loss of consciousness, and we prefer to use Natus EC2 EEG conductive adhesive paste for reusable electrodes, because it has an optimal cost–benefit ratio in terms of recording performance and time consumption compared to collodion. Furthermore, it does not require to move the patient to a dedicated room with gas extractor hood to apply the EEG electrodes.

### Tips for performing cardiovascular autonomic function tests in the COVID-19 era

The patient who proved fit after undergoing the screening procedure is admitted to the laboratory room wearing a surgical face mask. Only the patient and the healthcare provider are admitted (no companions); however, this may vary depending on the dimensions of the room. Healthcare staff pay attention to maintain at least 1 m distance [5, 9] from the patient whenever possible. Some guidelines specify 2 m distance [14, 15]; however, it is not always possible to adopt this measure during CAFT, which requires a certain vicinity for setting up the equipment, guiding the patient through the maneuvers, and checking the correct execution of each test.

After collecting the medical history, the healthcare provider measures height, weight and arterial blood pressure (BP) with the sphygmomanometer on both arms. A disposable film is applied to the patient’s arm before applying the arm BP cuff.

The patient is then laid on the tilt table. This is a possible high-risk procedure due to the proximity between the patient and the healthcare professional, especially for non-self-sufficient patients. In such cases, assistance from more than one person may be required. We prefer to ask the help of the patient’s companion rather than employing another member of staff. As a matter of fact, this person had been already screened during the triage, and the staff member performing CAFT is protected by the PPE anyway. Subsequently, electrodes and other elements necessary for the recording of ECG, thoracic and abdominal breathing, and beat to beat BP monitoring (finger cuff) are applied to the patient. It is advisable to use disposable devices or reusable ones which can be sanitized with appropriate solutions. The hand is sanitized before applying the finger BP cuff. Afterwards, either an extra-large glove or a protective disposable film is used to wrap the patient’s hand to avoid possible contamination. Care should be paid not to cover the frontend unit to avoid possible overheating. Then the recording is started and the selected CAFT procedures are performed.

Particular caution has to be paid during the execution of the Valsalva maneuver, as it is an aerosol-generating procedure and requires the removal of the face mask. We perform the Valsalva maneuver with an open glottis, by asking the patient to blow into a mouthpiece connected to a sphygmomanometer to maintain a forced and stable expiratory pressure for 15 s. In order to do so, the mouthpiece contains a hole which allows the leakage of air, which is therefore dispersed in the surrounding ambient air. Furthermore, the patient might have difficulty maintaining the mouth perfectly adhering to the mouthpiece and this may also cause leakage of forced expiratory air. We advise the patient to hold the mouthpiece with the hand of the free arm making a fist around it close to the mouth, thus reducing the risk of further dissemination of aerosols. We ask the patient to wear a glove during this procedure which is then disposed of; otherwise the hand is immediately sanitized afterwards. Caution should be taken not to close the hole for air leakage on the mouthpiece with the fist. Generally, we ask the patient to perform the Valsalva maneuver three times. Nevertheless, the patients should perform the least possible number of attempts and no more trials should be pursued once a successful maneuver (i.e., correctly executed with regard to the entity, maintenance and duration of the respiratory effort and the presence of the expected BP response) has been achieved. All reusable mouthpieces must be treated as potentially infectious and sanitized singularly immediately after use.

The deep breathing test instead could be performed by the patient while wearing the surgical face mask, thus reducing the risk of possible contamination. In accordance with our standard protocol, we usually perform 10 breathing cycles; however, if correctly executed, five are sufficient to assess heart rate variability induced by respiration. The specialized technician performing CAFT checks that the patient correctly follows the instructions.

While the patient performs the isometric exercise, we advise wrapping the cuff/hand dynamometer in a disposable glove before handing it to the patient to hold.

At the end of the assessment, after the patient has left, we allow at least a 1-h downtime for air exchange and return to baseline conditions. This time was calculated by doubling the passive air change rate of our laboratory, for additional precaution. This time frame includes the laboratory cleaning and sanitization procedures that are required at the end of the examination. In addition, it is a precaution to ensure proper social distancing.

**Fig. 1:**
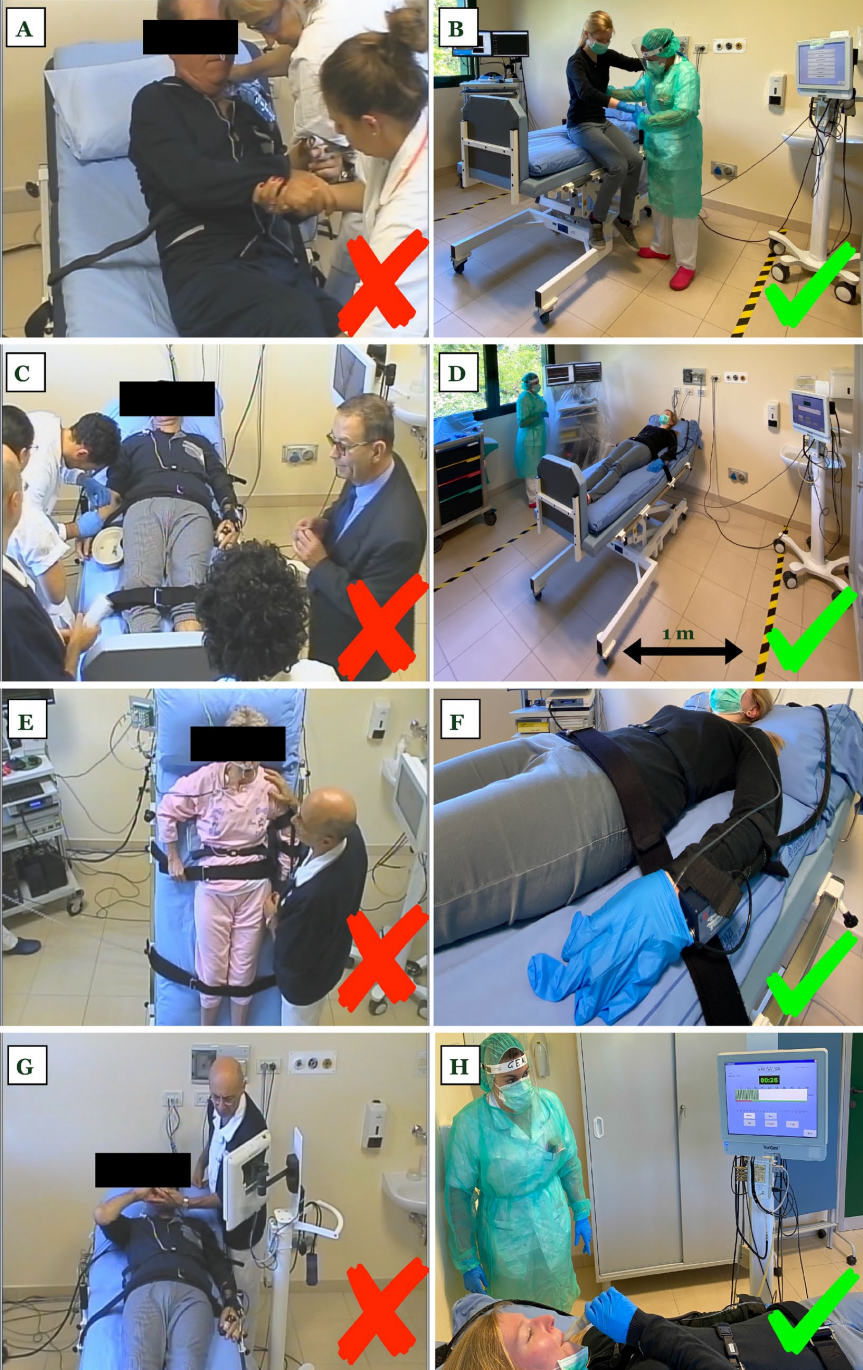
Practical tips to avoid risky contact during CAFT. Helping the patient getting on and off the tilt table before (**a**) and during (**b**) the COVID-19 era. No bedside teaching is allowed (**c**). Only the patient and one staff member are allowed inside the laboratory room, where colored tape delineates the area within 1 m from the tilt table; note that all equipment is located outside this area (**d**). The staff should remain at least 1 m apart for the whole duration of the protocol unless strictly necessary (**e**). The finger cuff is applied after having sanitized the finger; an extra-large glove is then worn above it (**f**). During the Valsalva maneuver, the staff member avoids close proximity (**g**) and checks the correct execution from 1 m distance (**h**). The use of disposable mouthpieces is strongly recommended

Figure 1 summarizes the main critical steps during the performance of CAFT and the suggested solutions to overcome them.

## Conclusions

The COVID-19 pandemic has deeply shaken our healthcare systems, which so far have responded with incredible professionalism and flexibility. These same qualities will be required when planning new strategies to safely reschedule laboratory activities.

Recently the American Autonomic Society published a position statement regarding autonomic function testing in the COVID-19 pandemic [2]. While this article provides general guidance, our paper aims to provide some useful practical tips for autonomic laboratories performing CAFT in the COVID-19 era. Our article is based on our experience at a tertiary referral center for autonomic disorders in an area that was massively affected at the beginning of the pandemic, and on national and international current regulations. Our paper intends to represent a guidance only and not a universal set of recommendations. This is not a substitute for good clinical practice and local rules.

Autonomic units have to face several other challenges, including advice for the management of patients affected by chronic autonomic disorders, some of which may be at increased risk of contracting the infection or of a more severe course [16].

Sharing information about personal professional experience and keeping up to date on scientific developments are the keys to increasing our knowledge on COVID-19 and its consequences on neurological diseases. Our final aim is to keep providing the best possible care to our patients in these difficult times.
